# Dataset of genome identification and characterization of microsatellite markers loci in *Atriplex atacamensis* and *Atriplex deserticola*

**DOI:** 10.1016/j.dib.2019.104258

**Published:** 2019-08-09

**Authors:** Francisco Correa, Jorge Pérez-Díaz, Pamela Rojas, Claudia Torres, Manuel Paneque, Boris Sagredo, Adriana Bastías

**Affiliations:** a Facultad de Ciencias de la Salud, Instituto de Ciencias Biomédicas, Universidad Autónoma de Chile, Providencia, Santiago de Chile, Postal Code: 7500000, Chile; b Facultad de Ciencias Agronómicas, Universidad de Chile, La Pintana, Santiago de Chile, Postal code: 8820000, Chile; c Laboratorio de Biotecnología y Recursos Naturales, Instituto de Investigaciones Agropecuarias (INIA) CRI Rayentué, Rengo, Postal code: 2940000, Chile

**Keywords:** Atriplex, SSR, Microsatellite, Molecular markers, Simple sequence repeat

## Abstract

In this work, we partially sequenced genomes of two Atriplex species (*A. deserticola* Phil. and *A. atacamensis* Phil.), using Illumina technology (Hiseq 2500 paired-end system) and *de novo* assembly strategy. Raw data of *A. deserticola* and *A. atacamensis* are available from NCBI-Bioproject, PRJNA495747 and PRJNA495763 accessions, respectively. A total of 127086 and 134984 microsatellite or simple sequence repeat (SSR) markers were identified within *A. deserticola* and *A. atacamensis* genomic DNA, respectively. In addition, predicted putative genes in *A. deserticola* and *A. atacamensis* sequences are also presented in this article.

**Table:** Specifications Table

Subject area	Plant biology
More specific subject area	Plant genomic sequencing and bioinformatic
Type of data	Tables, graphs and figures from data from Next-Generation Sequencing, SSR markers and predicted genes of *A. deserticola* and *A. atacamensis*
How data was acquired	Partial genome sequencing was realized using Illumina HiSeq 2500 Sequencer. The clean reads were *de novo* assembled to generate contigs, using SOAPdenovo software. SSR markers were determined using MIcroSAtellite identification software. Putative genes were predicted with AUGUSTUS software.
Data format	Raw, filtered and analyzed data
Experimental factors	Leaves of *A. deserticola* and *A. atacamensis,* DNA extraction, sequencing
Experimental features	Genomic DNA was extracted from leaves of *Atriplex deserticola* and *Atriplex atacamensis* plants with the DNeasy Plant Mini Kit. The paired-end library was sequenced using Illumina HiSeq 2500 Sequencer (Macrogen Inc., Seoul, Korea).
Data source location	Samples were collected at the Las Cardas Agricultural Experimental Field Station, University of Chile, (Coquimbo, Chile), S30°16′54.108″, O:71°15′16.486″
Data accessibility	Raw data of *A. deserticola* and *A. atacamensis* are available from NCBI-Bioproject, PRJNA495747 (https://www.ncbi.nlm.nih.gov/bioproject/?term=PRJNA495747) and PRJNA495763 (https://www.ncbi.nlm.nih.gov/bioproject/?term=PRJNA495763) accessions, respectively.
Related research article	Thiel, T., Michalek, W., Varshney, R.K. and Graner A. (2003) Exploiting EST databases for the development and characterization of gene-derived SSR-markers in barley *(Hordeum vulgare* L.*).* Theor Appl Genet. 106(3): 411–422. https://doi.org/10.1007/s00122-002-1031-0.

**Table:** 

**Value of the data** • The identified genomic sequences are an important resource for genetic, genomic and evolutionary studies and will Atriplex conservation and breeding programs. • These newly developed microsatellite loci of *A. atacamensis* and *A. deserticola* can be useful for molecular studies, including the construction of linkage maps, QTL mapping and association mapping for these two species. • Predicted genes of *A. deserticola* and *A. atacamensis* can be compared to the known genome sequence of similar or closely related organisms in order to identify any key similarities or differences and/or to investigate the function of a particular gene. • The partial sequences of genes, and SSR markers from high throughput next generation sequencing (NGS) of *A. atacamensis* and *A. deserticola* genomic DNA, constitute the first platform to undertake genetic and molecular studies of these plant species.

## 1 Data

Raw partial genome sequencing data *for A. deserticola and A. atacamensis* was produced by *de novo* sequencing using a HiSeq 2500 System - Illumina. The data was then quality trimmed, filtered and assembled (assembly statistics are present in Table 1, Table 2).

**Table 1:** Data of number of Atriplex (*A. deserticola* and *A. atacamensis*) reads obtained by NGS before and after cleaning.

Atriplex sp.	Before cleaning		After cleaning		% total reads
	Total reads	GC (%)	Total reads	GC (%)	
*A. deserticola*	876,957,994	35	803,100,062	36	91
*A. atacamensis*	874,405,882	35	761,570,465	35	87

**Table 2:** Data on contig measurements that were assembled by SOAPdenovo2 software after high-quality reads.

	*A. deserticola*	*A. atacamensis*
Contigs	274,412	302,895
N50	1256	1229
Count ≥ N50	68816	76561
Max contig	29233	28538
Min contig	500	500
Total length	310,278,579	338,284,897
Average contig	1131	1117

**Fig. 1:**
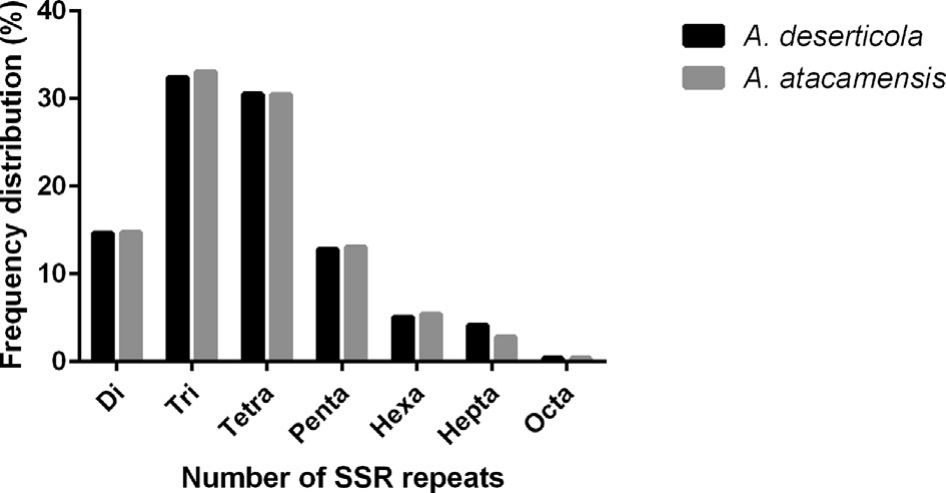
**Frequency distribution of SSR loci by motif length on the assembled genomic sequences of *A. deserticola* and *A. atacamensis.*** The graph is based on a total of 127086 and 134984 SSR markers detected in non-redundant genomic DNA of *A. deserticola* and *A. atacamensis*, respectively. Di, tri, tetra, penta, hexa, hepta and octa refer to dinucleotides, trinucleotides, tetranucleotides, pentanucleotides, hexanucleotides, heptanucleotides and octanucleotides, respectively.

Dinucleotide to hexanucleotide repeat microsatellite sequences were identified for *A. deserticola* and *A. atacamensis* (Table 3). However, only SSRs with a repeat motif size ranging from 2 to 8 bp and a length ≥12 bp were considered. This includes dinucleotide repeats ≥6, trinucleotide repeats ≥4, and tetra-, penta-, hexa-, hepta- and octanucleotide repeats ≥3. We analyzed the distribution of *A. deserticola* and *A. atacamensis* SSRs data with regard to motif length, type and number of repeats (Table 4, Table 5, Fig. 1). Primer pairs were designed from flanking sequences of di-to octanucleotide microsatellites of *A. deserticola* and *A. atacamensis* (S1 and S2 Tables).

**Table 3:** Microsatellite (SSRs) searches of *A. deserticola* and *A. atacamensis* using MIcroSAtellite identification.

Category	*A. deserticola*	*A. atacamensis*
Total number of sequences examined	274412	302895
Total size of examined sequences (bp)	310,278,579	338,284,897
Total number of identified SSRs	127086	134984
Number of SSR containing sequences	98939	104235
Number of sequences containing more than 1 SSR	35721	38381

**Table 4:** Distribution of microsatellites di-to octonucleotide motifs in the assembled genomic DNA of *A. deserticola* and *A. atacamensis*.

Motif length	N° loci identified		Frequency (%)		Density (SSR/Mb)	
	*A. deserticola*	*A. atacamensis*	*A. deserticola*	*A. atacamensis*	*A. desertícola*	*A. atacamensis*
Di	18605	19915	14.64	14.75	59.96	58.87
Tri	41175	44645	32.40	33.07	132.71	131.98
Tetra	38789	41160	30.52	30.49	125.02	121.67
Penta	16298	17645	12.82	13.07	52.53	52.16
Hexa	6448	7313	5.07	5.42	20.78	21.62
Hepta	5278	3806	4.15	2.82	17.01	11.25
Octa	493	500	0.39	0.37	1.59	1.48
Total/mean	127086	134984	100	100	409.60	399.03

**Table 5:** Summary of the frequency of SSRs from *A. deserticola* and *A. atacamensis* with different numbers of tandem repeats.

Motif length	Largest SSRs		Highest %	
	*A. deserticola*	*A. atacamensis*	*A. deserticola*	*A. atacamensis*
Di	(CT)35	(AG)36	AT/AT (7.78)	AT/AT (7)
Tri	(AAT)29	(TTA)29	AAT/ATT (13.31)	AAT/ATT (12.07)
Tetra	(TTTA)14	(TAAA)13	AAAT/ATTT (11.35)	AAAT/ATTT (10.14)
Penta	(AAAAT)8	(TAAAT)10	AACTG/AGTTC (2.61)	AAAAT/ATTTT (2.36)
Hexa	(TAAAAA)8	(TTTATT)8	AAAAAT/ATTTTT (0.84)	AAAAAT/ATTTTT (0.78)
Hepta	(AAACCCT)12	(CTAAACC)12	AAATAAT/ATTATTT (1.82)	AAACCCT/AGGGTTT (0.58)
Octa	(GTCAATGT)5	(GAGAAGA)6	AAAAAAAT/ATTTTTTT (0.1)	AAAAAAAG/CTTTTTTT (0.11)

**Fig. 2:**
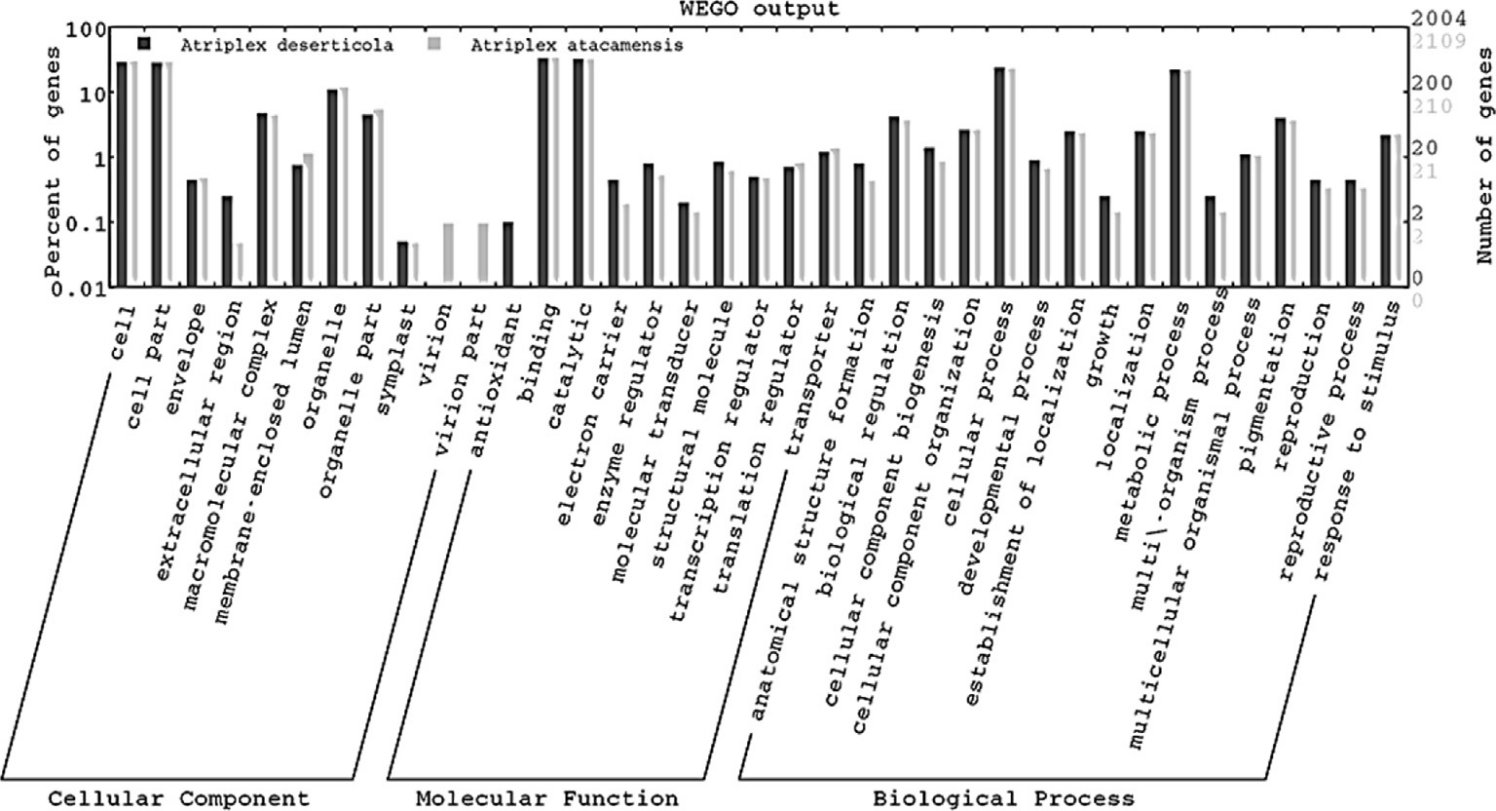
Gene Ontology classification of the predicted *A. deserticola* and *A. atacamensis* ORFs. The classification was predicted according to cellular component, molecular function and biological process using WEGO software.

The contig and singleton *A. deserticola* and *A. atacamensis* genomic sequences were analyzed by AUGUSTUS software [1], [2] using *A. thaliana* as a model organism to predict putative genes (Table 6). For functional annotation, the potential coding regions data were analyzed by WEGO [3], leading to consistent gene annotations, gene names, gene products and Gene Ontology (GO) numbers (Fig. 2 and S3 Table).

**Table 6:** Putative genes found in partial sequences of *A. deserticola* and *A. atacamensis* predicted by Augustus software.

Species	Total genes	Complete sequence	Partial sequence
*A. deserticola*	2443	372	2071
*A. atacamensis*	2581	360	2221

## 2 Experimental design, materials, and methods

### 2.1 Plant material and DNA extraction

Samples of *A. deserticola* and *A. atacamensis* were collected at the Las Cardas Agricultural Experimental Field Station, University of Chile, (Coquimbo, Chile). Genomic DNA (approximately 100 mg) was extracted from leaves of *A. deserticola* and *A. atacamensis* plants with the DNeasy Plant Mini Kit (Qiagen Inc., Valencia, CA, USA), following the manufacturer's protocols. DNA quality and quantity were checked by agarose gel electrophoresis and spectrophotometric measurement of UV absorption at wavelengths of 260 and 280 nm and absorbance ratios of 260/280 and 260/230, using an Infinitive M200Pro Nanoquant (Tecan Group US, Inc., Morrisville, NC, USA).

### 2.2 Next-generation sequencing

The Illumina paired-end library was prepared with the Illumina TruSeq DNA PCR-Free350 bp Library Preparation Kit (Illumina, San Diego, CA, USA). The paired-end library was sequenced using Illumina HiSeq 2500 Sequencer (Macrogen Inc., Seoul, Korea) using the TruSeq rapid SBS kit or Truseq SBS Kit v4 (Illumina, San Diego, CA, USA). The read sequence length was 126 nts from one end of the fragment to the other.

The sequence quality of raw genomic data was assessed using FastQC v0.11.5 (http://www.bioinformatics.babraham.ac.uk/projects/fastqc). The data was quality trimmed and filtered using PRINSEQ v0.20.4 (http://prinseq.sourceforge.net/) [4]. Reads containing more than 5% of unknown nucleotides, low-quality reads (those with more than 50% bases with Q-value ≤ 20) and unpaired reads were discarded. Short reads (<35 bp) were removed from the filtered data.

Raw data of *A. deserticola* and *A. atacamensis* are available from NCBI-Bioproject, PRJNA495747 and PRJNA495763 accessions, respectively.

The clean reads were *de novo* assembled to generate contigs, using SOAPdenovo 127mer (http://soap.genomics.org.cn/soapdenovo.html) [5] with an optimized k-mer length of 91, as calculated by KmerGenie software [6].

### 2.3 In silico identification of putative SSRs and primer design

We analyzed perfect and imperfect SSRs. The contig sequences obtained in FASTA files were screened with a repeat motif size range of 2–6 bp and a length of >12 bp. This included dinucleotide repeats ≥6, trinucleotide repeats ≥4, and tetra-, penta-, hexa-, hepta- and octanucleotide repeats ≥3, using MIcroSAtellite identification software [7], [8]. The program allows for direct primer design using PRIMER 3 [9] by searching for microsatellite repeats and primer annealing sites in the flanking regions (S1 and S2 Tables).

### 2.4 Putative *A. deserticola* and *A. atacamensis* gene prediction

Putative genes were predicted with AUGUSTUS software [1], [2], analyzing contig and singleton genomic sequences from *A. deserticola* and *A. atacamensis.* The program is based on a hidden Markov model and is used for the *ab initio* prediction of protein coding genes in eukaryotic genomes. *Arabidopsis thaliana* (L.), Heynh. was used as the model organism. WEGO (Web Gene Ontology Annotation Plot) software was then used to functionally annotate potential coding sequences or predicted genes [3]. A manual inspection of the predicted genes was performed to maximize the accuracy of gene prediction. The genes encoding predicted proteins were scored using the NCBI non-redundant (NR), Uniprot, and GO database. Matches were selected with the value e ≤ 1xe-5 and with 40% sequence identity.
